# Medical students’ attitudes and perspectives regarding novel computer-based practical spot tests compared to traditional practical spot tests

**Published:** 2013-09-30

**Authors:** Buddhika Wijerathne, Geetha Rathnayake

**Affiliations:** 1 Department of Forensic Medicine, Rajarata University of Sri Lanka, Sri Lanka; 2 Teaching Hospital Anuradhapura, Sri Lanka

## Abstract

**Background:**

Most universities currently practice traditional practical spot tests to evaluate students. However, traditional methods have several disadvantages. Computer-based examination techniques are becoming more popular among medical educators worldwide. Therefore incorporating the computer interface in practical spot testing is a novel concept that may minimize the shortcomings of traditional methods. Assessing students’ attitudes and perspectives is vital in understanding how students perceive the novel method.

**Methods:**

One hundred and sixty medical students were randomly allocated to either a computer-based spot test (*n*=80) or a traditional spot test (*n*=80). The students rated their attitudes and perspectives regarding the spot test method soon after the test. The results were described comparatively.

**Results:**

Students had higher positive attitudes towards the computer-based practical spot test compared to the traditional spot test. Their recommendations to introduce the novel practical spot test method for future exams and to other universities were statistically significantly higher.

**Conclusions:**

The computer-based practical spot test is viewed as more acceptable to students than the traditional spot test.

## Background

The use of computer-based methods of assessment in medicine is not a new idea.^1^,^2^ Computer-based assessment has been in development since the early 1960s.^3^ Its acceptability as a means of student assessment is well established.^4^,^5^ Many universities around the world are now frequently using computers to deliver, mark, and analyze student assessments. 6 Several studies have shown that computer-based tests perform well and student surveys have shown that such tests are frequently more popular than traditional tests.^7^,^8^

Most of the medical faculties in Asia currently use the traditional practical spot test examination as a multi-station spot test that is accompanied by objects, specimens, photographs or laboratory test results, along with the appropriate question for each station.

Studies that account for the disadvantages related to this method are extremely rare. The most apparent disadvantages that we observed were the need for students to move from one station to the next and the need for many invigilators and technical staff to observe and guide the students.

It is vital to revolutionize the traditional practical spot test by incorporating new technology. Introducing a computer-based interface for spot test stations is a novel concept that offers several advantages, including incorporation of high-quality images, drawings, multimedia^9^ and simulations.^10^ It is important to evaluate students’ acceptance of this novel method as a high-quality, student-friendly, and efficient practical spot test is essential to future curriculum development.

## Methods

**Figure 1:**
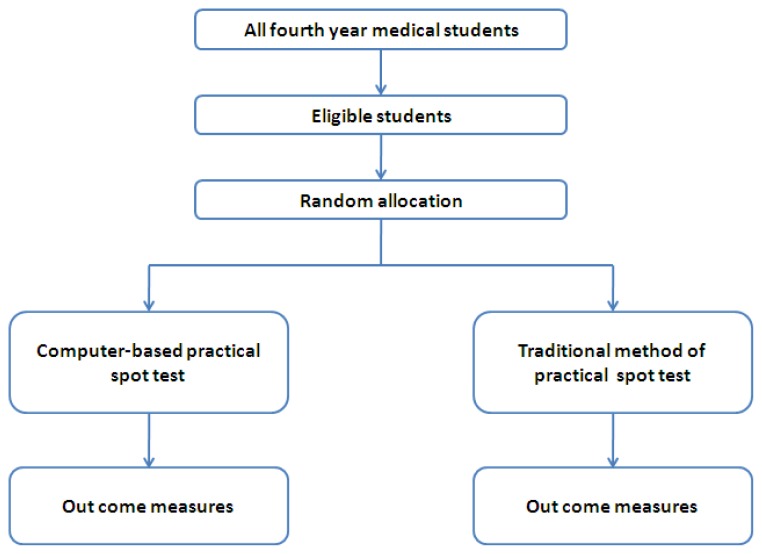
Study design

The study design is presented in Figure 1. Approval for the study was obtained from the university. Our target population included all fourth-year medical students. All students were given a series of lectures on basic mechanical injuries. One hundred and sixty students who participated in the consecutive lecture series and who gave their informed written consent were included in the study. The purpose of the study was explained to the students. The students who consented to participate in the study were individually assigned an identifying number. They were randomly allocated to two groups: either the computer-based practical spot test group (*n*=80) or the traditional practical spot test group (*n*= 80).

Both groups were given a practical spot test consisting of the same validated twenty questions. The tests lasted one hour and took place at the same time. All students were provided with the same paper-based answer sheet including numbered questions and blank spaces for answering.

### The computer-based practical spot test group (n =80)

**Figure 2:**
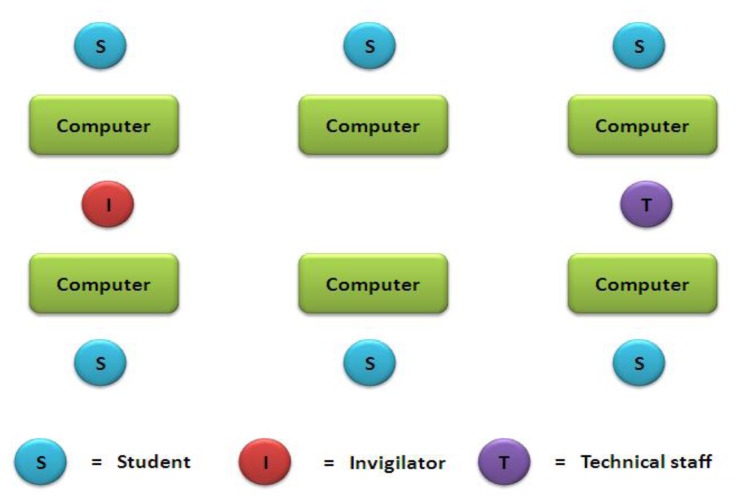
Computer-based practical spot test arrangement

The students in this group were given the computer-based spot test. Each student was seated at a separate computer throughout the exam. Twenty spot test stations were offered with a computer-generated presentation created by commonly used software. Spot tests consisted of clearly detailed, high-quality photos, video clips or simulations. Each spot-test station lasted three minutes and was programmed to automatically change to next the spot test station. The question relevant to the spot station was shown on the same screen. Students could sit comfortably in front of the computer and answered all the spot stations displayed on the computer (Figure 2).

The instructor explained the method briefly to the students before they started answering questions. Two invigilators observed each session and one technical staff member was present to guide students.

### The traditional practical spot test group (n =80)

**Figure 3:**
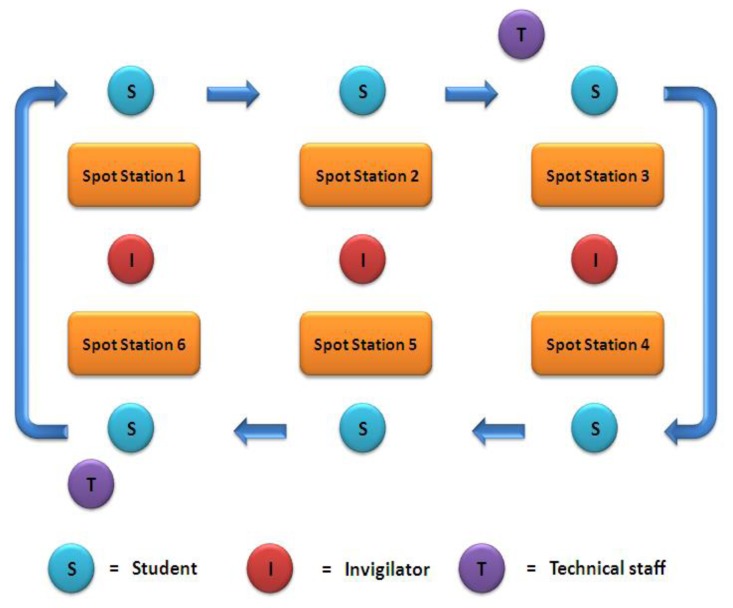
Traditional practical spot test arrangement

Twenty separate spot stations were arranged according to the questions. Stations consisted of photographs, objects or laboratory test results. Students commenced the test from the station where they were seated first and moved on to the next station at the bell which reminded them that the three minutes allocated to each station were over. Six invigilators observed each session and four technical staff members were present to guide the students (Figure 3).

Immediately after the spot test each student completed a questionnaire with questions about their attitudes towards the spot test method. The questions were divided into five pre-agreed categories as follows:

1. I was able to manage time efficiently for each spot station
2. I was able to recall facts efficiently during the spot test
3. I was able to maintain concentration during the spot test
4. I thought the setup was easy
5. I thought the setup was less stressful

A validated five-point Likert scale was used to assess student answers.

On the same questionnaire, the students’ perspectives regarding the spot test method were assessed with the following Yes/No type questions:

1. Do you recommend this method for future practical spot tests?
2. Do you recommend this method for practical spot tests in other universities?

Statistical analyses were done by using SPSS-20. Chi-square analyses were carried out to assess the association between variables. A probability of *p*<0.05 was considered statistically significant.

## Results

### Students’ attitudes

Tables 1 and 2 show the students’ ratings of their attitudes towards the spot test.

### Efficient time management for spot stations

Of the students who completed the computer-based practical spot test 70% (*n*=56) agreed that the novel method aided them in efficient time management, whereas 25% (*n*=20) agreed that the traditional method aided efficient time management.

### Recalling facts efficiently during the test

Of the students who completed the computer-based practical spot test 81.25% (*n*=65) agreed that the novel method aided them in recalling facts efficiently, where as 16.25% (*n*=13) agreed that the traditional method aided them in recalling facts efficiently.

### Maintaining concentration during the spot test

Of the students who completed the computer-based practical spot test 83.75% (n=67) agreed that the novel method facilitated maintaining concentration, whereas 40% (*n*=32) agreed that traditional method facilitated maintaining concentration.

### Simplicity of the setup

Of the students who completed the computer-based practical spot test 80% (*n*=64) agreed that the novel method was easy, whereas 35% (*n*=28) agreed that traditional method was easy.

### Stress during the spot test

Of the students who completed the computer-based practical spot test 71.25% (*n*=57) agreed that the novel method was less stressful, whereas 21% (*n*=11) agreed that the traditional method was less stressful.

### Students’ perspectives

Of the students who participated in the computer-based practical spot test 81.25% (*n*=65) recommended it for future exams. Only 25 %(*n*=20) of students recommended the traditional spot test method for future exams (Table 3).

Of the students who participated in the computer-based practical spot test 72.25% (*n*=57) recommended it for other medical faculties, whereas only 8.75% (*n*=07) students recommended the traditional spot test for other medical faculties (Table 4).

## Discussion

In the present study the students’ attitudes and perspectives regarding practical spot tests were subjectively assessed. Our approach to assess the educational intervention was student oriented. We employed the randomization process to promote similarity between the student groups in terms of baseline characteristics to avoid allocation bias. The study involved a relatively large number of participants, which maximized the efficiency of the study.

Today, with advancing technology, medical educators are facing different challenges than their forerunners in teaching and evaluating tomorrow’s medical students. Computer-based assessment methods have gained popularity but its use is highly variable among medical schools. The computer-based practical spot test is generally acceptable to students and they find it less threatening than conventional exams. The computer-based spot test can result in significant cost savings compared with traditional spot tests. It saves staff time in terms of supervision, preparation costs, and reduced labour. It is a suitable method for developing countries. However, it should be clear that computer-based assessment must not be seen as a “quick fix”.

The initial implementation of computer-based tests can be costly and time consuming. Staff who design and invigilate the computer-based method need to be trained in evaluation principles, test design, and information technology (IT) skills. Hardware and software used to deliver a spot test needs to be robust in order to avoid failure at crucial times during examinations. Students need to have basic and sufficient IT skills. Once that application has been setup, the cost of offering it to additional groups of students is relatively small.

Concerning student attitudes and perspectives, we only assessed several predefined criteria. There could be other negative and positive areas we overlooked in our assessment. Our study only assessed the attitudes of students related to the subject of forensic medicine. Future research should include testing with other subjects and address different student perspectives and attitudes.

Because of the popularity of computer-based testing, now seems a critical time to advance this line of research. Most universities are already beginning to introduce a wide range of computer-based systems, sometimes in an indiscriminate way. Planned and synchronized development is better than haphazard expansion. Computer-based tests are particularly suited to academic fields that are visually intensive, detail oriented, and difficult to conceptualize, such as complex biochemical processes or microscopic imaging.

## Conclusions

Students’ positive attitudes towards computer-based practical spot tests are higher than for the traditional spot test method. It appears, therefore, that the novel method is more acceptable to students than the traditional spot test. They recommended it for future spot tests and for use in other universities as a preferred practical spot-test method. Further studies are needed to assess whether computer-based practical spot tests may enhance student performance.

## Figures and Tables

**Table 1:** Students’ attitudes – computer-based practical spot test

Area rated subjectively	Strongly Agree		Agree		Undecided		Disagree		Strongly Disagree	
	M (%)	F (%)	M (%)	F (%)	M (%)	F (%)	M (%)	F (%)	M (%)	F (%)
**I was able to efficiently manage time for each spot station’**	4 (5)	17 (21.25)	14 (17.5)	21 (26.25)	7 (8.75)	6 (7.5)	5 (6.25)	4 (5)	2 (2.5)	0 (0)
**I was able to recall facts efficiently during the spot test**	17 (21.25)	25 (31.25)	8 (10)	15 (18.75)	2 (2.5)	3 (3.75)	4 (5)	5 (6.25)	1 (1.25)	0 (0)
**I was able to maintain concentration during the spot test**	17 (21.25)	19 (23.75)	11 (13.75)	20 (25)	2 (2.5)	3 (3.75)	1 (1.25)	5 (6.25)	1 (1.25)	1 (1.25)
**I thought the setup was easy**	14 (17.5)	12 (15)	15 (18.75)	23 (28.75)	3 (3.75)	11 (13.75)	0 (0)	1 (1.25)	0 (0)	1 (1.25)
**I thought the set up was less stressful**	10 (12.5)	14 (17.5)	9 (11.25)	24 (30)	6 (7.5)	6 (7.5)	5 (6.25)	4 (5)	2 (2.5)	0 (0)

**Table 2:** Students’ attitudes– traditional practical spot test

Area rated subjectively	Strongly Agree		Agree		Undecided		Disagree		Strongly Disagree	
	M (%)	F (%)	M (%)	F (%)	M (%)	F (%)	M (%)	F (%)	M (%)	F (%)
**I was able to efficiently manage time for each station**	3 (3.75)	2 (2.5)	8 (10)	7 (8.75)	4 (5)	7 (8.75)	11 (13.75)	18 (22.5)	13 (16.25)	7 (8.75)
**I was able to recall facts efficiently during the exam**	0 (0)	1 (1.25)	6 (7.5)	6 (7.5)	11 (13.75)	6 (7.5)	16 (20)	20 (25)	6 (7.5)	8 (10)
**I was able to maintain concentration during the exam**	10 (12.5)	2 (2.5)	12 (15)	8 (10)	4 (5)	6 (7.5)	4 (5)	17 (21.25)	9 (11.25)	8 (10)
**I thought the setup was Easy**	3 (3.75)	2 (2.5)	15 (18.75)	8 (10)	13 (16.25)	6 (7.5)	5 (6.25)	18 (22.5)	3 (3.75)	7 (8.75)
**I thought the set up was less stressful**	0 (0)	2 (2.5)	2 (2.5)	7 (8.75)	5 (6.25)	4 (5)	15 (18.75)	20 (25)	17 (21.25)	8 (10)

**Table 3:** Students’ recommendation for future practical spot tests

Group	Yes	No	Total
Computer-based practical spot test (*n*=80)	65	15	80
Traditional practical spot test (*n*=80)	20	60	80
Total	75	85	160

(χ^2^ = 50.82, df = 1, *p* = 0.005)

**Table 4:** Students’ recommendation for spot tests at other universities

Group	Yes	No	Total
Computer-based practical spot test (*n*=80)	57	23	80
Traditional practical spot test (*n*=80)	7	73	80
Total	75	85	160

(χ^2^ = 65.10, df = 1, *p* = 0.005)
